# Supplementary material for: Development and validation of animal variant classification guidelines to objectively evaluate genetic variant pathogenicity in domestic animals
Source: Front Vet Sci. 2024 Dec 5;11:1497817. doi: 10.3389/fvets.2024.1497817 (PMC11656590; doi:10.3389/fvets.2024.1497817)
Supplement: Supplementary file 1 [file Data_Sheet_1.DOCX]

Supplementary Material

# Supplementary Data

Suppl. Data S1. Overview of the systematic review strategy to identify pathogenic and benign variants, according to the PRISMA guidelines.

- Type of review: systematic review
- Primary research question: Which strategies are used in benchmark studies to identify pathogenic and benign/neutral variants?
- Search strategy:
  - 1. Database: PubMed
  - 2. Websites: https://www.varianteffect.org/veps
  - Inclusion criteria: all articles that are included should describe a selection strategy for pathogenic or benign variants to assess performance of a variant effect predictor
  - Exclusion criteria:
    - No mention of a selection strategy for benign and/or neutral variants
- Query string:

("missense"[Title/Abstract] OR "nonsense"[Title/Abstract] OR "in-frame"[Title/Abstract] OR "frameshift"[Title/Abstract] OR "splice site"[Title/Abstract]) AND ("benchmark" OR "performance") AND ("variant effect predictor"[Title/Abstract] OR "in silico"[Title/Abstract]) NOT ("cancer*" OR "tumor" OR "tumour") and date set to prior 2024-01

- Search validation procedure (check for bias):

The following reports should be retrieved:

1. Ghosh R, Oak N, Plon SE. Evaluation of in silico algorithms for use with ACMG/AMP clinical variant interpretation guidelines. Genome Biol. 2017 Nov 28;18(1):225. doi: 10.1186/s13059-017-1353-5. PMID: 29179779; PMCID: PMC5704597.
2. Thusberg J, Olatubosun A, Vihinen M. Performance of mutation pathogenicity prediction methods on missense variants. Hum Mutat. 2011 Apr;32(4):358-68. doi: 10.1002/humu.21445. Epub 2011 Feb 22. PMID: 21412949.

All aforementioned reports were found
