# Supplementary material for: Development and validation of animal variant classification guidelines to objectively evaluate genetic variant pathogenicity in domestic animals
Source: Front Vet Sci. 2024 Dec 5;11:1497817. doi: 10.3389/fvets.2024.1497817 (PMC11656590; doi:10.3389/fvets.2024.1497817)
Supplement: Supplementary file 2 [file Data_Sheet_2.DOCX]

Supplementary Material

Suppl. Data S2. Overview of the systematic review strategy to identify *in silico* tools, according to the PRISMA guidelines.

- Type of review: systematic review
- Primary research question:
  - Which variant effect predictors exist?
  - Which ones have an online user interface?
  - Which of the remaining ones can be used for animals?
- Search strategy:
  - 1. Database: PubMed
  - 2. Websites: https://www.varianteffect.org/veps
  - Inclusion criteria: all articles that are included should describe a tool that predicts the functional impact and accepts cat proteins, which is easily accessible (i.e., a web-tool where you can submit the variant immediately without a download)
  - Exclusion criteria:
    - No web-tool
    - Cannot be used for animals
    - Tool requiring a fee
- Query string:

("missense"[Title/Abstract] OR "nonsense"[Title/Abstract] OR "in-frame"[Title/Abstract] OR "frameshift"[Title/Abstract] OR "splice site"[Title/Abstract]) AND ("benchmark" OR "performance") AND ("variant effect predictor"[Title/Abstract] OR "in silico"[Title/Abstract]) NOT ("cancer*" OR "tumor" OR "tumour") and date set to prior 2024-01

- Search validation procedure (check for bias):

All tools reported in the veterinary literature should be retrieved based on these results. These were MaxEntScan, NNSplice, PolyPhen-2, SNAP, PROVEAN, SIFT, MutPred, PredictSNP, CADD, Meta-SNP and Condel. All tools were found.
