# Supplementary material for: Development and validation of animal variant classification guidelines to objectively evaluate genetic variant pathogenicity in domestic animals
Source: Front Vet Sci. 2024 Dec 5;11:1497817. doi: 10.3389/fvets.2024.1497817 (PMC11656590; doi:10.3389/fvets.2024.1497817)
Supplement: Supplementary file 3 [file Data_Sheet_3.DOCX]

Supplementary Material

# Supplementary Data

Suppl. Data S3. Explanatory comments of criteria in the Variant Classification Guidelines.

**PVS1**The group finds it important to specify that evidence for loss-of-function as a known mechanism can be based on the same or other species, but if it is across species, it is also important to evaluate whether it is likely that the function of the gene is similar. The reason to include this “function” part is e.g., that some genes are pseudogenes in one species and not in another, something which is more likely to occur if species are distantly related. If a gene is expected, or has been shown to be functional in the species studied, this criterion can be used. From the description, it is clear that this criterion applies to variants that disrupt gene function. This is not always easy to assess: e.g., the presence of alternative transcripts can complicate interpretation and some transcripts are tissue-specific.

**PS1**If a variant results in an amino-acid change that has already been shown to be pathogenic, this criterion applies. If a variant results in a **different** amino-acid than the one that has been shown to be pathogenic, it is not this criterion that applies, but PM2.

**PS2**It is important to evaluate this vertically throughout the pedigree: parents should test negative for the variant. If the parents are not available or were not tested, this criterion cannot be used.

**PS3**No additional explanation.

**PS4**The group finds it difficult to confidently decide on which odds ratio (OR) is sufficiently high and at the same time to be of practical use as a cut-off. Deciding on which cut-off is appropriate has a lot of consequences: large sample sizes can lead to significant results with relatively low point estimates and vice versa. In human studies, an OR of 5 has been suggested to be an appropriate cut-off. Studies in animals suggest a similar cut-off would also be appropriate. An explanation on how to calculate the various ORs is provided (Suppl. Data S5*)*. There are two important remarks for this criterion:

- this criterion cannot be used for *de novo* variants as there is no true “enrichment” in cases: it typically will only occur once. For *de novo* variants, the PS2 criterion should thus be used.
- it is important to ensure an adequate sample size is available. A power analysis should be performed.

**PS5**Practically, this implies that in one or more pedigrees, the variant is present in a disease-causing state in affected family members and that the variant is not present in a disease-causing state in healthy individuals. When evaluating this criterion, it is important to take into account that the litter size can vary across species and even across breeds within a species as this influences the size of the pedigree that can reasonably be expected.

**PM1**No additional explanation.

**PM2**The group finds it important to specify that this also involves across species.

**PM3**Several remarks are important in this criterion. Firstly, both variants should be reported jointly to allow an assessment of the association. Secondly, it may be difficult to assess the effect of each individual variant: some only jointly have an effect. Thirdly, sometimes phenotypes, even in the same gene, can be inherited in an autosomal dominant or an autosomal recessive manner, depending on the variant. Practically, this means that an additional variant, next to a pathogenic variant that turns out to cause a phenotype in a dominant manner, might erroneously be labelled "pathogenic". The final remark is that, although this might be a valid criterion, it should not be weighted too high for final classification of a variant.

**PM4**It is important to emphasize that this criterion does **not** apply for variants in non-conserved regions.

**PP1/BP1**
The criterion was actually worded as “*Cross-species alignment to determine whether a variant is conserved (= supportive for pathogenic) or not conserved (=supportive for benign).* This criterion can support benign or pathogenic classification depending on the answer. It does not refer to *in silico* tools that predict the effect of a variant, because that falls under PP3 or BP4. It does refer to 1/ a cross-species sequence alignment to evaluate conservation of nucleotides or amino-acids (depending on what is relevant for that specific variant) **and** 2/ a cross-species check of the potential role of the variant based on other information, like ClinVar (e.g., if the variant has been described in ClinVar, is it considered a pathogenic or benign variant there).

**PP2**The group finds it important to specify that this also involves across species.

**PP3/BP4**

Multiple **different** software tools support prediction. While it is difficult to objectively quantify what the optimal number of tools is as this also depends on how similar the tools are and their availability, the group decided that at least two different tools, if available, have to be used. Only if **all** tools lead to the same conclusion (i.e., when two are used, both say deleterious or both say harmless), the criterion is accordingly fulfilled. If tools are conflicting, the criterion is not fulfilled. An overview of the tools that support the use of non-human data per variant type is provided in Table 6. Furthermore, we recommend checking benchmark studies to select the most accurate tools and to keep in mind that for some variant types, *in silico* tools have a poor accuracy.

**PP4**This criterion only applies when (i) most patients test positive for a pathogenic variant in that gene; (ii) the patient has a well-defined syndrome with little overlap with other clinical presentations. One example is Duchenne muscular dystrophy, in which a variant in the *DMD* gene is expected. Only very limited genetic heterogeneity can thus be present for this criterion to be valid. When one observes the phenotype, one has immediately one, at most few, gene(s) in mind.

**BS1**Caveat: the presence of phenocopies for common phenotypes (e.g., epilepsy) can mimic lack of segregation among affected individuals.

**BS2/BS3**No additional explanation.

**BP1**see PP1.

**BP2/BP3/BP5/BP6**

No additional explanation.
