# Supplementary material for: Development and validation of animal variant classification guidelines to objectively evaluate genetic variant pathogenicity in domestic animals
Source: Front Vet Sci. 2024 Dec 5;11:1497817. doi: 10.3389/fvets.2024.1497817 (PMC11656590; doi:10.3389/fvets.2024.1497817)
Supplement: Supplementary file 4 [file Data_Sheet_4.DOCX]

Supplementary Material

# Supplementary Data

Suppl. Data S4. Reporting recommendations and quality checks when publishing disease-associated variants.

In what follows, we provide some general recommendations, based on difficulties repeatedly encountered during the review process.

**Statistical set-up and calculations**

To describe genotype-phenotype associations, usually odds ratios and relative risks are used. To allow a check of these data, a 2x3 table describing the distribution of genotypes across the phenotypical categories is important. Furthermore, as various methods to perform the calculations exist, we recommend a clear description of what is exactly calculated. For odds ratios, we provide examples and terminology in Suppl. Data S4.

**Variant description**

Variant descriptions are often incomplete and do not contain version numbers. As new annotations and reference genomes are constantly published, it can be difficult to decipher exactly which variant is described. Aside from descriptions according to the accepted nomenclature, we in addition suggest to provide the neighbouring amino acids and/or nucleotides (what is most appropriate) as well as this facilitates identification of the important variant. This can for example be done in a figure describing cross-species alignment and/or electropherograms.

**Mode of inheritance, disease penetrance and genetic heterogeneity**

The distribution of genotypes across the phenotypical categories should allow a substantiated proposal for a mode of inheritance and furthermore detail whether penetrance (defined as the probability to identify the phenotype, given a genotype) is expected to be complete or reduced, as well as whether genetic heterogeneity is expected. These three items are important during the evaluation of several criteria and should thus be mentioned in the manuscript.

**Population study**

While allele frequency has been removed from the AVCG as criterion influencing classification, all group members agreed that population studies are of paramount importance when new variants are reported or already published variants investigated further. Population studies provide an overview of how widespread a variant is, i.e., whether it is case-, family-, breed-specific or common overall. Furthermore, when breeding strategies are developed, the prevalence of a disease-
