# Supplementary material for: Development and validation of animal variant classification guidelines to objectively evaluate genetic variant pathogenicity in domestic animals
Source: Front Vet Sci. 2024 Dec 5;11:1497817. doi: 10.3389/fvets.2024.1497817 (PMC11656590; doi:10.3389/fvets.2024.1497817)
Supplement: Supplementary file 5 [file Data_Sheet_5.DOCX]

Supplementary Material

# Supplementary Data

Suppl. Data S5. Clarification of methods to calculate odds ratios.

|  | Genotypes | | |
| --- | --- | --- | --- |
|  | Vt/Vt | Wt/Vt | Wt/Wt |
| Cases | a | b | c |
| Controls | d | e | f |

With a – f representing the number of individuals in each cell.

Several methods exist to calculate odds ratios (ORs). We specify the following two:

1/ allelic OR: this requires calculation of allelic frequencies and hence collapsing of the table

|  | Allele | |
| --- | --- | --- |
|  | Vt | Wt |
| Cases | 2 x a + b = g | 2 x c + b = h |
| Controls | 2 x d + e = i | 2 x f + e = j |

Based on this table, the OR is calculated as:

$$OR =\frac{(g \times j)}{(i \times h)}$$

2/ genotype OR: this requires a specification of a mode of inheritance and collapsing of the genotypes accordingly. In more detail,

- for an autosomal recessive mode of inheritance, assuming Vt/Vt is affected:

|  | Genotype | |
| --- | --- | --- |
|  | Vt/Vt | Wt/Wt + Wt/Vt |
| Cases | a | b + c = h |
| Controls | d | e + f = j |

Based on this table, the OR is calculated as:

$$OR =\frac{(a \times j)}{(d \times h)}$$

- for an autosomal dominant mode of inheritance, assuming Wt/Vt and Vt/Vt are affected:

|  | Genotype | |
| --- | --- | --- |
|  | Wt/Vt + Vt/Vt | Wt/Wt |
| Cases | a + b = g | c |
| Controls | d + e = i | f |

Based on this table, the OR is calculated as:

$$OR =\frac{(g \times f)}{(i \times c)}$$

Remark:

When one of the cells is zero, the OR becomes zero as well or is not defined. One can apply at that moment a correction by adding 0.5 to every cell. In what follows, we provide an example, based on the WNK4:c.2899C>T variant, which follows an autosomal recessive mode of inheritance, with the following distribution of genotypes over cases and controls:

|  | Genotypes | | |
| --- | --- | --- | --- |
|  | Vt/Vt | Wt/Vt | Wt/Wt |
| Cases | 43 | 0 | 0 |
| Controls | 0 | 22 | 69 |

- For the allelic OR, the table collapses to:

|  | Allele | |
| --- | --- | --- |
|  | Vt | Wt |
| Cases | 43 x 2 = 86 | 0 |
| Controls | 22 | 69 x 2 + 22 = 160 |

As the OR is undefined in this example, we add 0.5 to every cell count:

|  | Allele | |
| --- | --- | --- |
|  | Vt | Wt |
| Cases | 86.5 | 0.5 |
| Controls | 22.5 | 160.5 |

The allelic OR now has a value of 1,234.067

- For an autosomal recessive mode of inheritance, the genotype OR table has the following values:

|  | Genotype | |
| --- | --- | --- |
|  | Vt/Vt | Wt/Wt + Wt/Vt |
| Cases | 43 | 0 |
| Controls | 0 | 91 |

As the OR is undefined in this example, we add 0.5 to every cell count:

|  | Genotype | |
| --- | --- | --- |
|  | Vt/Vt | Wt/Wt + Wt/Vt |
| Cases | 43.5 | 0.5 |
| Controls | 0.5 | 91.5 |

The genotype OR now has a value of 15,921.
