# Supplementary material for: Development and validation of animal variant classification guidelines to objectively evaluate genetic variant pathogenicity in domestic animals
Source: Front Vet Sci. 2024 Dec 5;11:1497817. doi: 10.3389/fvets.2024.1497817 (PMC11656590; doi:10.3389/fvets.2024.1497817)
Supplement: Supplementary file 7 [file Table_2.DOCX]

Suppl. Table S2. Overview of the original American College of Medical Genetics and Genomics (ACMG) guidelines and summary of changes.

| ACMG name | AVCG name | Criterion |
| --- | --- | --- |
| PVS1 | **PVS1** | Null variant (nonsense, frameshift, canonical ±1 or 2 splice sites, initiation codon, single or multiexon deletion) in a gene where LOF is a known mechanism of disease. |
| PS1 | PS1 | Same amino acid change as a previously established pathogenic variant regardless of nucleotide change. |
| PS2 | **PS2** | *De novo* (both maternity and paternity confirmed) in a patient with the disease and no family history. |
| PS3 | PS3 | Well-established *in vitro* or *in vivo* functional studies supportive of a damaging effect on the gene or gene product. |
| PS4 | PS4 | The prevalence of the variant in affected individuals is significantly increased compared with the prevalence in controls. |
| PM1 | **PM1** | Located in a mutational hot-spot and/or critical and well-established functional domain (e.g., active site of an enzyme) without benign variation. |
| PM2 | NA | Absent from controls (or at extremely low frequency if recessive) (Table 6) in Exome Sequencing Project, 1000 Genomes Project, or Exome Aggregation Consortium. |
| PM3 | PM3 | For recessive disorders, detected in *trans* with a pathogenic variant. |
| PM4 | PM4 | Protein length changes as a result of in-frame deletions/insertions in a nonrepeat region or stop-loss variants. |
| PM5 | **PM2** | Novel missense change at an amino acid residue where a different missense change determined to be pathogenic has been seen before. |
| PM6 | NA | Assumed *de novo*, but without confirmation of paternity and maternity. |
| PP1 | **PS5** | Cosegregation with disease in multiple affected family members in a gene definitively known to cause the disease. |
| PP2 | PP2 | Missense variant in a gene that has a low rate of benign missense variation and in which missense variants are a common mechanism of disease. |
| PP3 | **PP3** | Multiple lines of computational evidence support a deleterious effect on the gene or gene product (conservation, evolutionary, splicing impact, etc.) |
| PP4 | PP4 | Patient’s phenotype or family history is highly specific for a disease with a single genetic etiology. |
| PP5 | NA | Reputable source recently reports variant as pathogenic, but the evidence is not available to the laboratory to perform an independent evaluation. |
| BA1 | NA | Allele frequency is >5% in Exome Sequencing Project, 1000 Genomes Project, or Exome Aggregation Consortium. |
| BS1 | NA | Allele frequency is greater than expected for disorder. |
| BS2 | BS2 | Observed in a healthy adult individual for a recessive (homozygous), dominant (heterozygous), or X-linked (hemizygous) disorder, with full penetrance expected at an early age. |
| BS3 | BS3 | Well-established *in vitro* or *in vivo* functional studies show no damaging effect on protein function or splicing. |
| BS4 | BS1 | Lack of segregation in affected members of a family. |
| BP1 | NA | Missense variant in a gene for which primarily truncating variants are known to cause disease. |
| BP2 | BP2 | Observed in *trans* with a pathogenic variant for a fully penetrant dominant gene/disorder or observed in *cis* with a pathogenic variant in any inheritance pattern. |
| BP3 | BP3 | In-frame deletions/insertions in a repetitive region without a known function. |
| BP4 | **BP4** | Multiple lines of computational evidence suggest no impact on gene or gene product (conservation, evolutionary, splicing impact, etc.). |
| BP5 | BP5 | Variant found in a case with an alternate molecular basis for disease. |
| BP6 | NA | Reputable source recently reports variant as benign, but the evidence is not available to the laboratory to perform an independent evaluation. |
| BP7 | BP6 | A synonymous (silent) variant for which splicing prediction algorithms predict no impact to the splice consensus sequence nor the creation of a new splice site AND the nucleotide is not highly conserved. |

In the first column, the original ACMG name is reported. The second column depicts whether the criterion was removed (NA), altered (**AVCG criterion name in in bold**) or remained unchanged (AVCG criterion name in normal typeset).
