# Supplementary material for: Development and validation of animal variant classification guidelines to objectively evaluate genetic variant pathogenicity in domestic animals
Source: Front Vet Sci. 2024 Dec 5;11:1497817. doi: 10.3389/fvets.2024.1497817 (PMC11656590; doi:10.3389/fvets.2024.1497817)

Supplementary Material

Suppl. Figure S1. PRISMA 2020 flow diagram for new systematic reviews which included searches of databases, registers and other sources for selection strategies of benign and pathogenic variants.

.

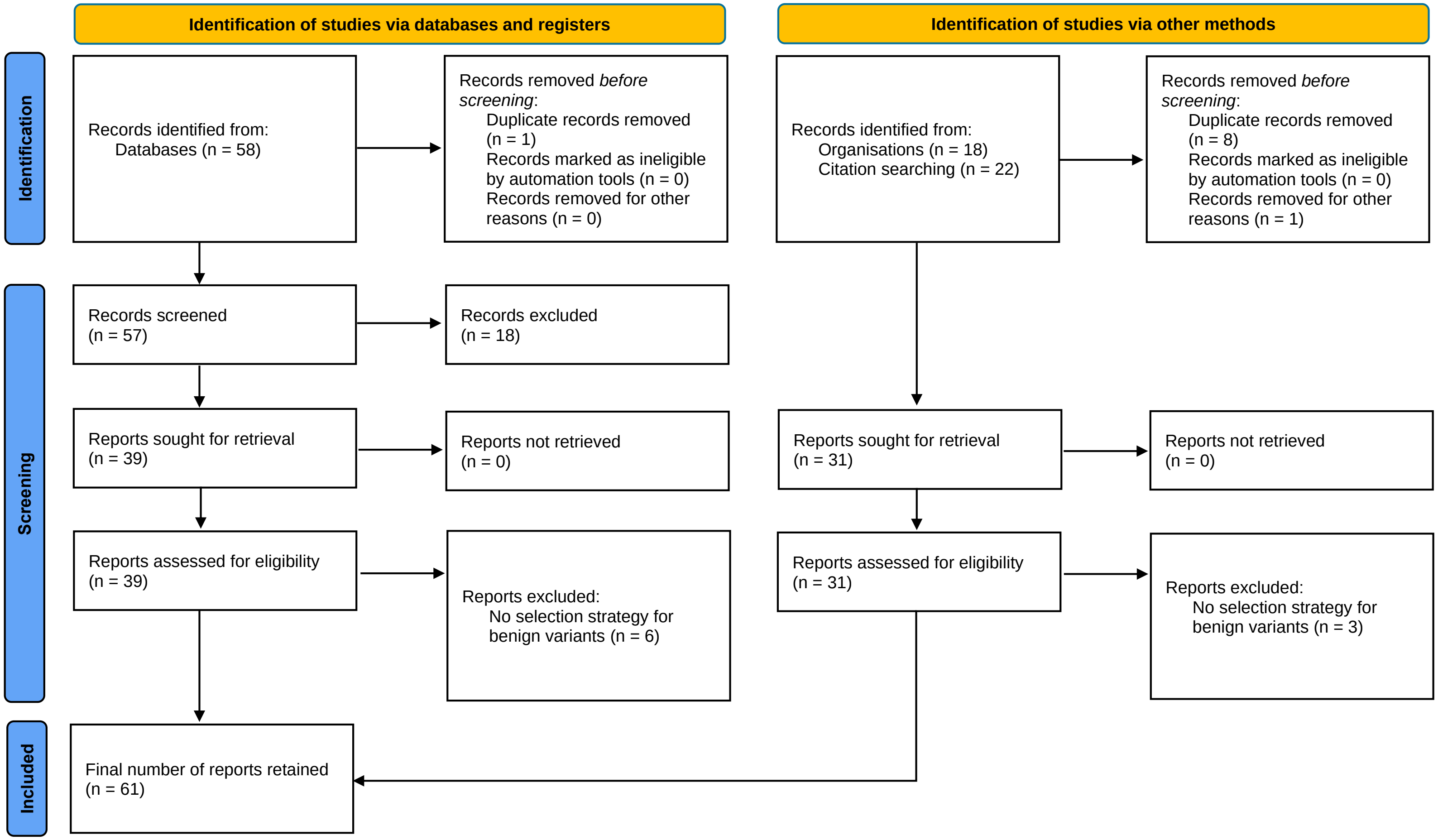

Suppl. Figure S2. PRISMA 2020 flow diagram for new systematic reviews which included searches of databases, registers and other sources for selection strategies of *in silico* tools.

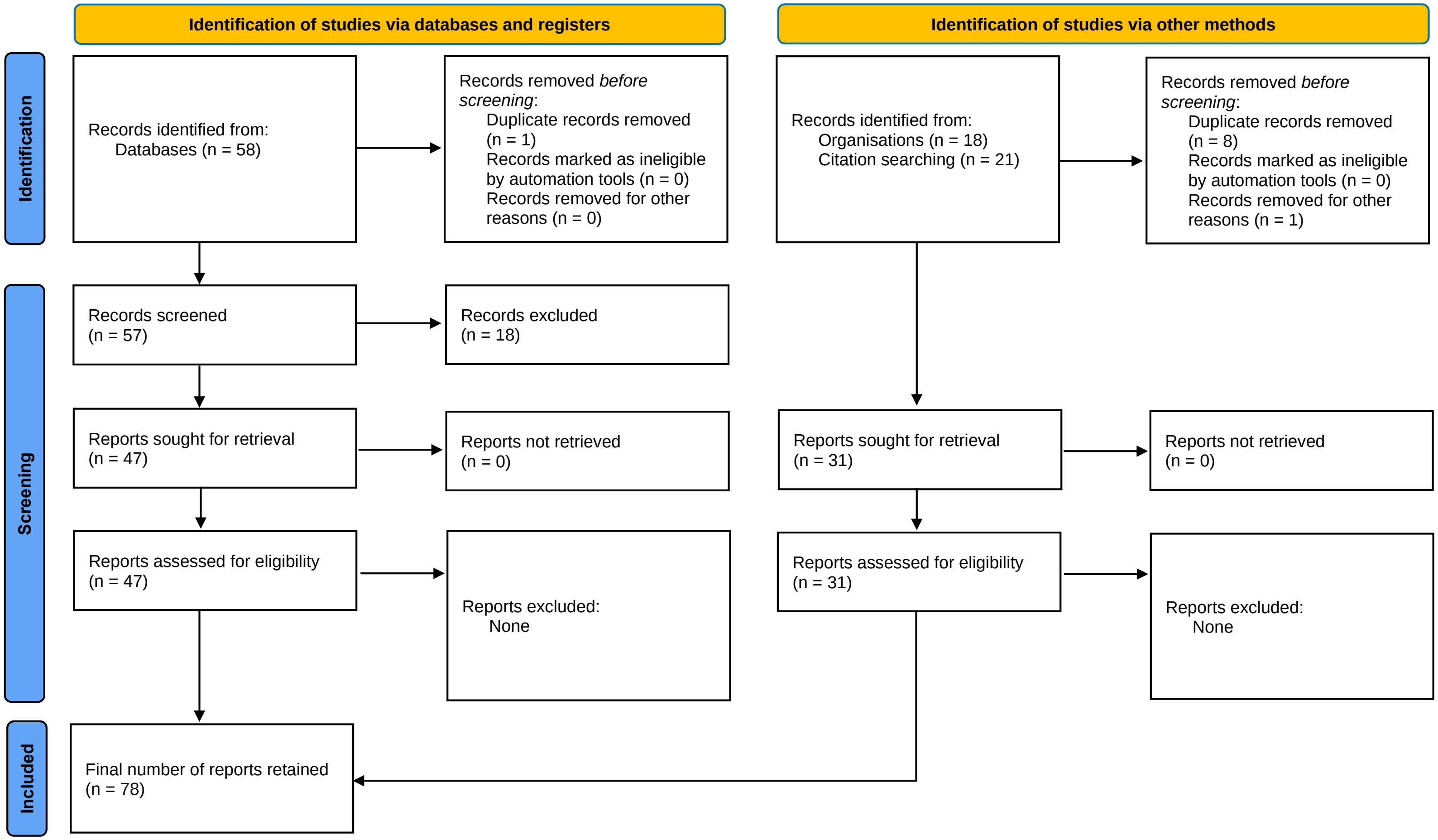

Supplement: Supplementary file 15 [file Image_1.pdf]
